# Vex-seq: high-throughput identification of the impact of genetic variation on pre-mRNA splicing efficiency

**DOI:** 10.1186/s13059-018-1437-x

**Published:** 2018-06-01

**Authors:** Scott I. Adamson, Lijun Zhan, Brenton R. Graveley

**Affiliations:** Department of Genetics and Genome Sciences, Institute for Systems Genomics, UConn Health, Farmington, CT USA

## Abstract

Understanding the functional impact of genomic variants is a major goal of modern genetics and personalized medicine. Although many synonymous and non-coding variants act through altering the efficiency of pre-mRNA splicing, it is difficult to predict how these variants impact pre-mRNA splicing. Here, we describe a massively parallel approach we use to test the impact on pre-mRNA splicing of 2059 human genetic variants spanning 110 alternative exons. This method, called variant exon sequencing (Vex-seq), yields data that reinforce known mechanisms of pre-mRNA splicing, identifies variants that impact pre-mRNA splicing, and will be useful for increasing our understanding of genome function.

**Electronic supplementary material:**

The online version of this article (10.1186/s13059-018-1437-x) contains supplementary material, which is available to authorized users.

## Background

One of the main goals of personalized medicine is to understand how genetic variations between individuals impact health. Genetic variants can impact health in a number of different ways, one of which is through altering pre-mRNA splicing efficiency. Alternative splicing is a process that is important for regulatory function and a primary source of proteome diversity in humans [1]. Perturbations in splicing have also been shown to contribute to a number of different diseases [2, 3]. These splicing changes can manifest themselves through interrupting well-known interactions between the spliceosome and splicing elements, including the 3′ and 5′ splice sites, pyrimidine tract, or branchpoint sequences. However, splicing can also be perturbed by disrupting other sequences known to modulate splicing. Exonic splicing enhancers and silencers (ESEs and ESSs), as well as intronic splicing enhancers and silencers (ISEs and ISSs), are examples of splicing regulatory elements that can be perturbed and result in different splicing outcomes. Modulation of these splicing regulatory elements has been shown to be disease associated (for a review see [4]). Thus, understanding how both intronic and exonic variants impact splicing not only provides insights into the mechanisms of splicing, but also is important to understand the basis of certain genetic diseases.

Identifying variants that impact splicing regulatory elements and their splicing consequences are difficult to detect using conventional poly(A)+ RNA-seq alone because the variants are often spliced out of the mature mRNA. A number of different studies have aimed to address this issue. One approach has been the pursuit of deciphering the “splicing code” using computational techniques such as deep learning [5–7]. While these studies have yielded useful knowledge about splicing and do have predictive power, experimental confirmation of the behavior of these variants has been limited and the predictions are not perfect. Other groups have pursued the use of random sequences to understand the splicing code; however, it is hard to integrate datasets with contextual transcriptome information (i.e., CLIP) when studying the splicing behavior of random sequences [8]. A more recent study tested a number of exonic disease-associated variants in parallel using a mini-gene system [9]. The approach was to observe the allelic ratio of reference to variant in a plasmid pool, and compare with the ratios observed from splicing outcomes. This approach is useful for studying exonic variants but is unable to test intronic variants. Here we present a method that address some of these shortcomings using a barcoding approach called Variant exon sequencing (Vex-seq). Vex-seq is capable of testing many exonic and flanking intronic variants for the same exon simultaneously.

## Results

**Fig. 1:**
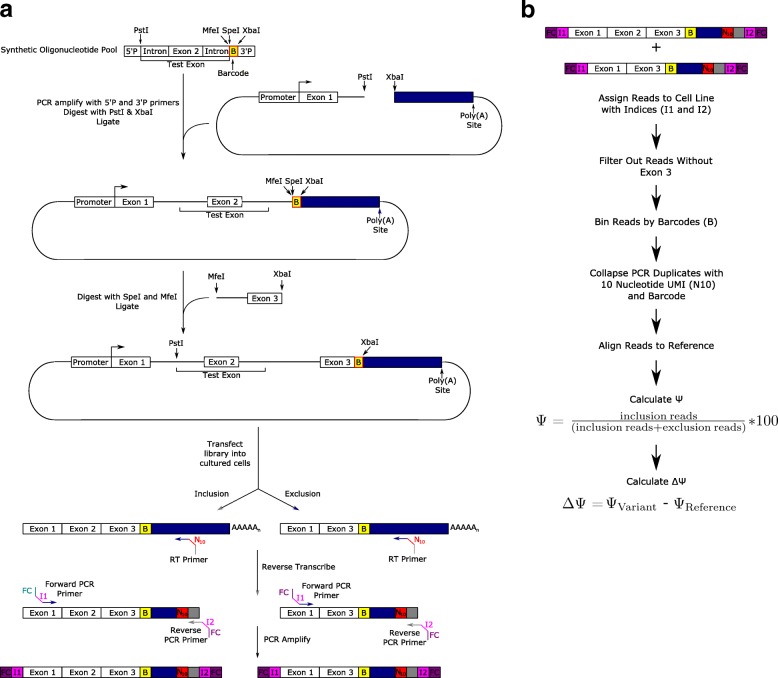
Assembly of test exon and experimental design. **a** The test exon and flanking introns are subcloned into a reporter plasmid in a two-step process, such that the barcode designating the sequence is near the end of the transcript. Once these plasmids are transfected into cultured cells, a transcript will be produced that may not contain the variant itself, but does contain the barcode (**b**) uniquely associated with the variant tested. A ten-nucleotide UMI (N10) is attached during the reverse transcription step to collapse PCR duplicates downstream. Illumina flow cell binding sequences (*FC*) and indexes (I1 and I2) are attached via primers during PCR and the resulting DNA is sequenced on a MiSeq platform. **b** Data analysis pipeline for splicing results

We set out to develop a high-throughput reporter system to determine the impact of genomic variants on pre-mRNA splicing. Our general approach is to generate a library of test exons flanked by two common constitutive exons (Fig. 1). The library was introduced into tissue culture cells followed by RT-PCR and sequencing to determine the splicing frequency of the test exon. Importantly, the reporters also contain a barcode sequence that serves as an identifier of which exon was present in the reporter so that it is possible to associate the pre-mRNA of origin in cases where the test exon was skipped.

We first designed a pool of 2059 variants spanning 110 exons with reference, consensus splice site, and mutated splice site control sequences for each exon. To ensure reproducibility, each variant exon was associated with at least three unique eight-nucleotide barcodes. Common primer sequences and restriction enzyme sites were also added for proper library construction. We included a minimum of 50 bases of the upstream intron, which should be adequate to include the majority of branchpoints [10], as well as the exon itself and at least 20 bases of downstream intron. This allowed for construction of test exons up to 97 nucleotides in length. Alternative exons between the size of 68 and 97 nucleotides were randomly selected from Ensembl GRCh37.p13 annotations and variants from the ExAC database were intersected with the selected exons and their flanking intron sequences [11].

We amplified the oligonucleotide pool by PCR (Additional file 1: Table S1). This product was then subcloned into a modified version of the splicing reporter plasmid pcAT7-Glo1 in between the first intron and the 3′ UTR to generate a 1˚ library. Then restriction sites in between the barcode and the end of the test sequence were used to subclone in the second part of the second intron and third exon from the original plasmid (Fig. 1a). This results in a plasmid that encodes a transcript containing the first exon and part of the first intron of the globin gene, the test sequence, followed by the second intron and final exon of the reporter transcript, ending with the barcode and the 3′ UTR. We refer to this final library pool as the 2˚ library.

**Fig. 2:**
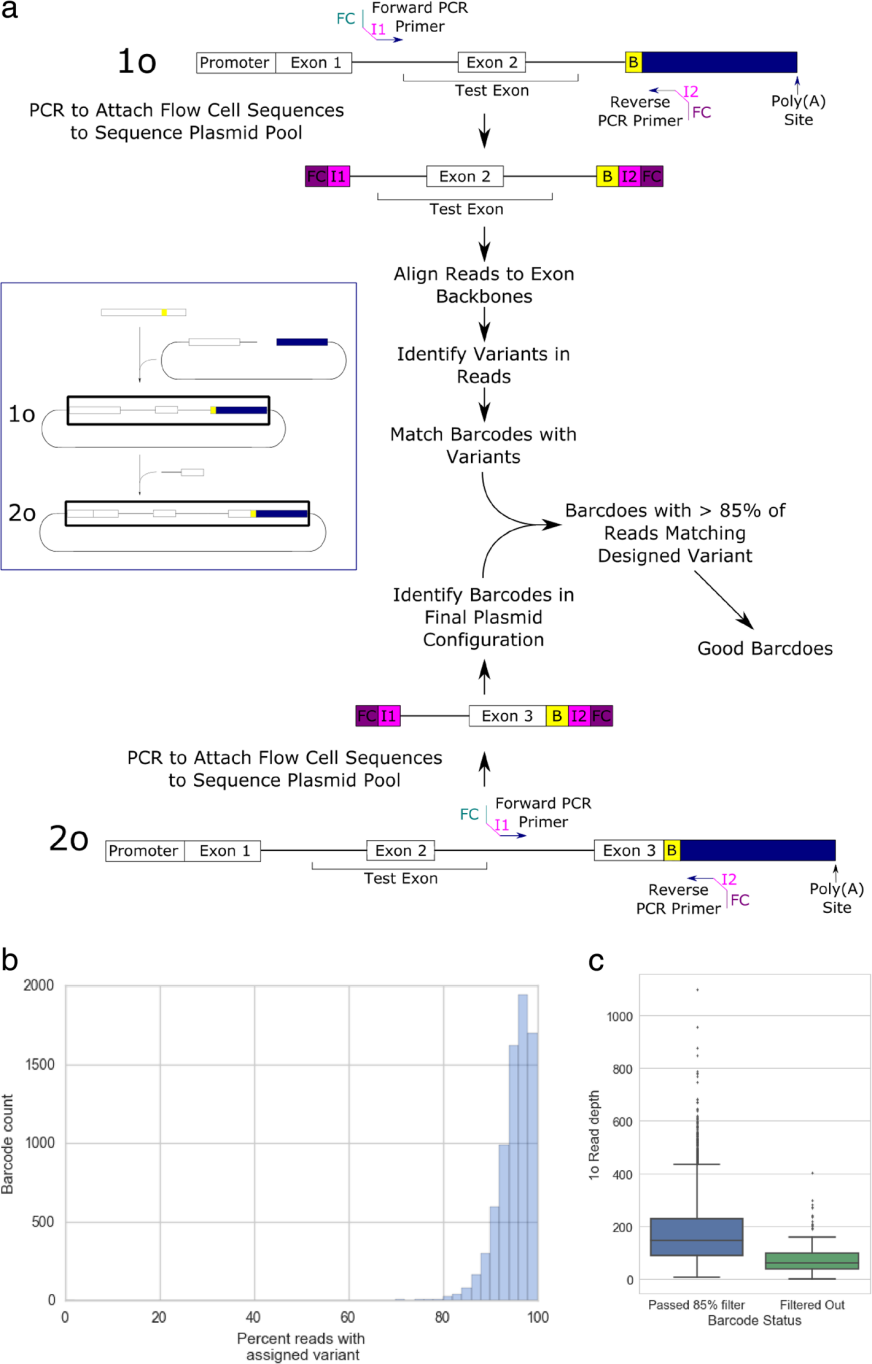
Quality control for plasmid integrity. **a** Quality control pipeline for plasmid integrity. Amplicon sequencing of the 1o and 2o plasmid configurations are done through PCR to attach Illumina flow cell binding sequences (*FC*) and indexes (I1 and I2). Poor quality barcodes are then filtered out by identification of reads not containing variants and excluding barcodes with less than 85% of reads containing the correct variant. **b** A histogram of the barcodes with the percentage of reads with correct variant identified. **c** Box plots of 1˚ library read depth for barcodes included and excluded from further analysis

In order to ensure that the variants are associated with the correct barcode, the 1˚ and 2˚ libraries were sequenced using paired end amplicon sequencing (Fig. 2a). The results from sequencing the 1˚ library show that the majority of barcodes are correctly associated with the correct variant (Fig. 2b). Barcodes excluded from the analysis due to having too few (less than 85%) of the correct variant reads associated with it only make up about 1.8% of the barcodes tested. Barcodes that were filtered out of the analysis also tended to have a lower read depth, suggesting that this may be related to the reason for their higher error rate (Fig. 2c). We are also able to measure a misassignment rate of 4.59% using this plasmid sequencing technique. In order to ensure that the library contained a good diversity of sequences, we calculated a skew ratio between the 10th and 90th percentile of read depth for each barcode as done previously to check library diversity [12]. A skew ratio for the library established was calculated to be 5.5, which is considered adequate. We conclude that despite some misassignment, the plasmid pool is robust enough to be used to study variant changes in splicing.

The 2˚ library was then transfected into K562 and HepG2 cell lines in biological triplicate. cDNA was then synthesized from the RNA isolated from the cells using a mini-gene specific primer, a ten-nucleotide random sequence which serves as a unique molecular identifier (UMI) and an Illumina Read 2 sequencing primer. PCR amplified the cDNA to attach the other necessary sequences for Illumina paired-end sequencing. The products were then sequenced on an Illumina MiSeq.

The data analysis pipeline uses custom python scripts to ensure that read 2 contains the third exon, the correct restriction site next to the barcode, and sorts the reads by barcode into bins. PCR replicate reads are collapsed into a single read using the UMI from the reverse transcription primer. The reads in each bin are then aligned using STAR to a reference specific to each variant [13]. Percent spliced in (PSI or Ψ) and change in PSI (ΔΨ) from the reference sequence are then calculated (Fig. 1b). The amplicon based paired-end sequencing reads contain an unambiguous splicing outcome for each amplicon, making Ψ and ΔΨ calculations straightforward from the alignment outputs alone.

**Fig. 3:**
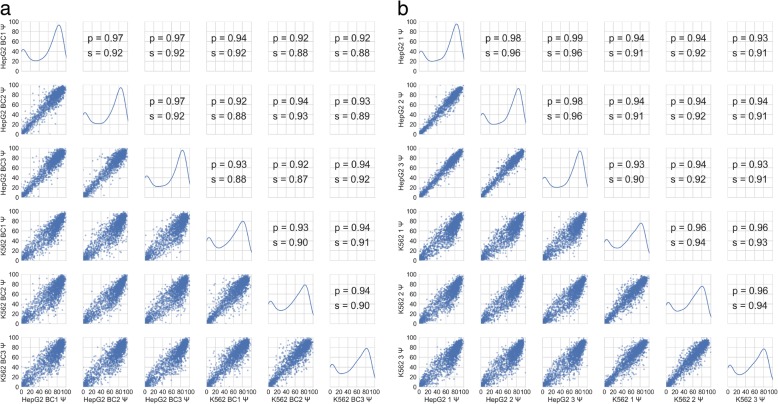
Behavior and reproducibility of splicing outcomes. **a** Scatter plots showing the behavior of Ψ for each barcode replicate of the same variant. These were averaged Ψ values of the barcode in each biological replicate. Spearman (*s*) and Pearson (*p*) correlations of Ψ are also shown. **b** Scatter plots showing the splicing behavior or Ψ for each variant in each biological replicate. Spearman (*s*) and Pearson (*p*) correlations of Ψ are also shown

To assess how similarly the barcodes associated with the same variant impacted the splicing behavior, we compared the Ψ value of the barcode replicates for each variant and reference exon and observed high correlations (Fig. 3a). To ensure that these splicing values were robust to biological variation, we also examined the correlations of variants between three biological replicates for HepG2 and K562 cell lines (Fig. 3b). HepG2 and K562 cell lines were chosen for these studies because of the wealth of potentially applicable ENCODE data associated with these cell lines and because they represent difference cell types and different *trans-*environments for splicing. These data show similarly high correlation values between biological replicates of the same cell lines, showing robustness to biological variation. These data also show that the splicing data from Vex-seq is robust to both technical and biological variation.

**Fig. 4:**
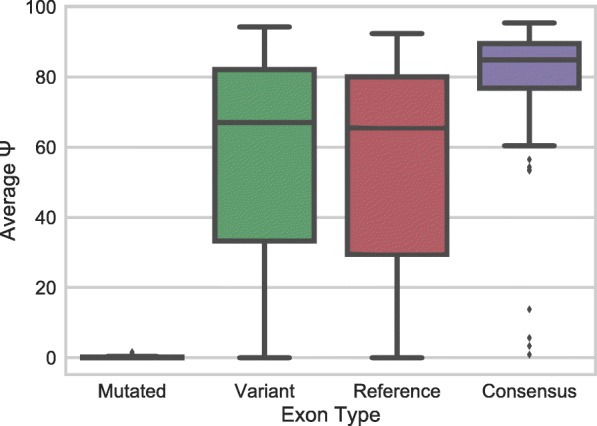
Splice site control sequences generally reflect expected splicing behavior. Boxplots of mean Ψ for each type of control and test sequences are shown. Mutated splice site controls contained mutated splice sites such that the 3′ splice sites were changed from AG to TC and the 5′ splice sites were changed from GT to CA. For the consensus splice site controls, the variants contained a 20-nucleotide pyrimidine tract, an AG at the 3′ splice site, and a consensus 5′ splice site of GTAAGT

In order to ensure that splicing behavior reflects what is known about splicing, we examined the Ψ of the mutated and consensus splice site controls relative to reference and variant exons. For the mutated splice site controls, both splice sites were mutated such that the 3′ splice sites were changed from AG to TC and the 5′ splice sites were changed from GT to CA. For the consensus splice site controls, the variants contained a 20-nucleotide pyrimidine tract, an AG at the 3′ splice site, and a consensus 5′ splice site of GTAAGT. The consensus and mutated splice site controls behave as expected with the mutated splice site controls displaying a low Ψ value and consensus splice sites having a higher Ψ value, while the variant and reference sequences are intermediate (Fig. 4). These are consistent with the expected splicing behaviors for these control sequences.

**Fig. 5:**
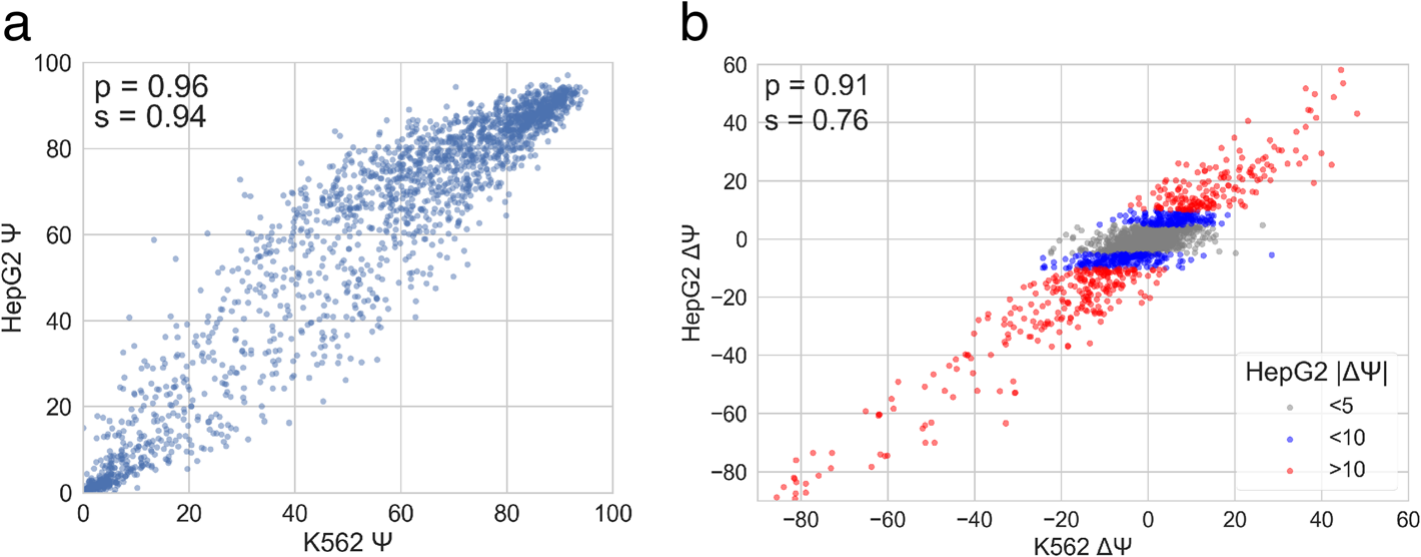
Similar behavior of splicing between K562 and HepG2 cell lines. **a** Correlation between Ψ values for each variant between K562 and HepG2 cell lines. **b** Correlation between ΔΨ values between K562 and HepG2 cell lines. Color coding highlights variants in which the HepG2 ΔΨ changes at different thresholds. Spearman (*s*) and Pearson (*p*) correlations are also displayed on each plot

Given the high correlation rates of Ψ values between the biological replicates of different cell lines, we sought to characterize this similarity further. Indeed, upon examining the correlation between the average Ψ value of each cell line, we observe a similar pattern (Fig. 5a). Additionally, when looking at changes in splicing (ΔΨ) we see a similar trend (Fig. 5b). In fact, 76.45% of variants agree in directionality of ΔΨ between cell lines. Furthermore, restricting this analysis to only include variants that have a ∣ΔΨ∣ > 5 or ∣ΔΨ∣ > 10 in HepG2, the agreement in directionality increases to 92.61% and 97.49%, respectively (Fig. 5b). This shows that although the Ψ of the reference exons can differ between cell lines, the directionality of most variant-induced changes in splicing studied in Vex-seq are cell type independent. However, there are exceptions to this trend, which could be due to the *trans* environment of each cell line or noise in the data.

**Fig. 6:**
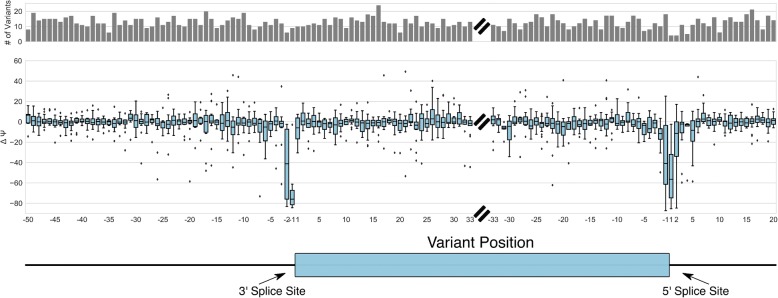
Distribution of variants tested and their impacts relative to splice sites. ΔΨ from both K562 and HepG2 cell lines is plotted for all variants relative to 3′ and 5′ splice sites. Fifty bases of upstream intron, 33 bases of exon proximal to the splice sites and 20 bases of downstream intron are shown. Above is a histogram showing the number of observations at each position

**Fig. 7:**
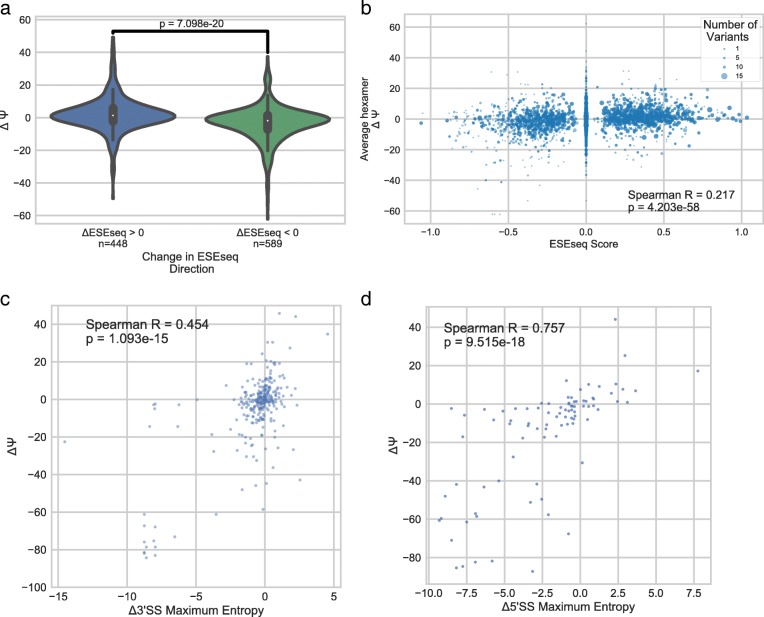
Analysis of potential mechanisms underlying splicing changes. **a** Violin plots showing how the directionality of a change in ESEseq score associates with splicing changes. *P* value is calculated using Mann-Whitney U-test. **b** Scatter plot demonstrating the relationship between the ESEseq score of each hexamer and the average ΔΨ of variance gaining (adding ΔΨ) or losing (subtracting ΔΨ) that hexamer. **c** Scatter plot showing the positive correlation between changes in 3′ splice site maximum entropy and ΔΨ. **d** Scatter plot showing the positive correlation between changes in 5′ splice site maximum entropy and ΔΨ. Spearman correlation coefficients and spearman correlation *p* values are shown in **c** and **d**

We next examined the changes in splicing efficiency (or ΔΨ) for each variant. Changes in splicing can be observed in many different positions relative to each of the splice sites; however, perturbations in the 3′ and 5′ splice sites typically result in a dramatic reduction of ΔΨ (Fig. 6). Many outliers can be observed changing ΔΨ negatively upstream of the 3′ splice site, which may correspond to changes in the pyrimidine tract or the branchpoint sequences. However, variants in core splicing sequences alone do not account for the full diversity of splicing variation observed from these data. Evidence of potential ESS and ESE regulatory elements can be observed within the exon, as variants in the exon are capable of inducing strong ΔΨ changes in either direction. We examined the role of ESEs and ESSs using ESEseq, a dataset which contains information about hexamers and their exonic splicing regulatory effect (ESEseq) [14]. We find that variants which gain ESEs or lose ESSs, as measured by change in ESEseq score, generally show an increase in ΔΨ, while ESS gain and ESE loss generally show a decrease (Fig. 7a). When examining changes in hexamer composition and the relationship of each hexamer with average changes in ΔΨ, we observe a weak but significant Spearman correlation with the ESEseq dataset (Fig. 7b). The weakness of this correlation is probably because the variation in our dataset was not designed to explore vast hexamer space, and many hexamer rankings are computed from few data points.

The impact of splice site strength has been well characterized and is known to impact splicing efficiency [15]. The impacts of changes on maximum entropy of 3′ and 5′ splice sites calculated by MaxEntScan can be visualized in Fig. 7c, d [15]. Intronic splicing regulatory elements have been typically studied downstream of the exon in question, which are outside or on the periphery of the context that Vex-seq currently has the capacity to study [16, 17]. However, we do still observe intronic variants changing splicing (particularly upstream of the 3′ splice site) that are outside of the conventionally measured effects of changes in 3′ and 5′ splice site strength. As many of the variants that do impact splicing are upsteam of the exon, yet outside the window studied for 3′ splice site strength, we examined whether these might disrupt branchpoint sequences. To do this, we used branchpointer, a machine learning program, to predict branchpoint probabilities of the reference and variant branchpoints [18]. Surprisingly, the majority (53 out of 84) of variants impacting splicing in this region were not predicted to impact maximum branchpoint usage probability. The variants that do affect branchpoint probability did not show any significant correlation with ΔΨ. We also did not identify any association between changes in RNA secondary structure around the splice sites and changes in splicing, which have been previously reported [19].

**Fig. 8:**
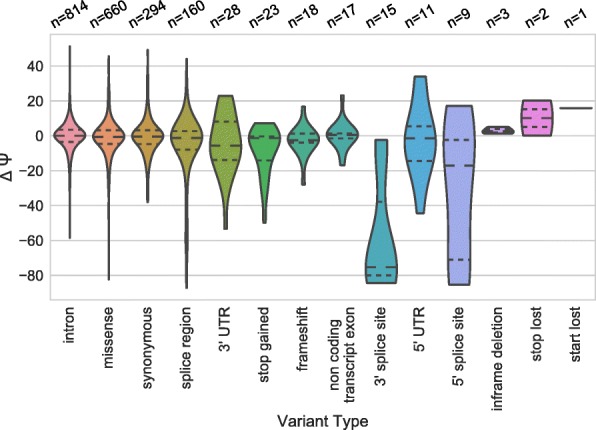
Variants classified by effect prediction and their impact on ΔΨ. Splice region classified by VEP is defined as being within one to three bases of the proximal exon, or three to eight bases of the proximal introns. The splice donor and acceptor annotations strictly refer to the dinucleotides downstream and upstream of the exon, respectively. The first reported annotation by VEP is displayed

To further characterize variants being studied and how they impact splicing we looked at other features, including effect predictions. Using Variant Effect Predictor (VEP) [20] annotations we characterized the variants and their impact on ΔΨ (Fig. 8). Annotations were selected based on the first reported annotation from VEP. The 5′ and 3′ splice site variants have the biggest negative impact on ΔΨ as expected. Intron, missense, synonymous, and splice region variants can also have a wide range of impacts on ΔΨ. This is consistent with previous findings about how missense and synonymous variants can change splicing inclusion levels [21]. It should also be noted that splice region variants alone do not account for many of the variants which changed splicing, consistent with the difficulty of predicting the impact of these variants based on impact annotations.

**Fig. 9:**
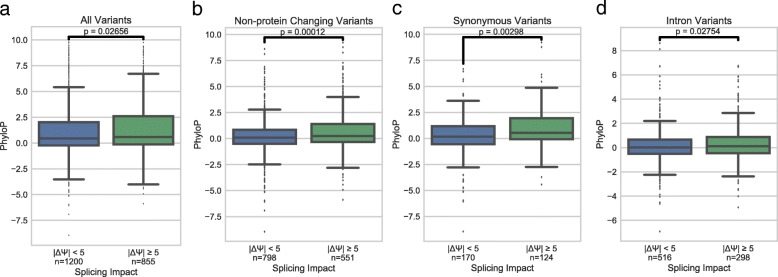
Conservation of variants with strong splicing impacts. **a** Boxplots showing the relationship of PhyloP and magnitude of ΔΨ for all variants. **b** Boxplots showing the relationship of PhyloP and magnitude of ΔΨ for variants without predicted protein coding annotations. **c** Boxplots showing the relationship of PhyloP and magnitude ΔΨ for synonymous variants. **d** Boxplots showing the relationship of PhyloP and magnitude of ΔΨ for intron variants. *P* values are calculated with the Mann-Whitney U-test. All variant effect predictions were performed by VEP and were classified by the first reported annotation

We were also interested in examining whether the variants that displayed the largest ΔΨ were more or less conserved than variants that had little impact on ΔΨ. We used 100-way vertebrate conservation scores from PhyloP to examine how variants with strong or weak impacts on splicing were conserved [22]. We observed that there is significantly more conservation in variants which tend to have a high impact on splicing (|ΔΨ| ≥ 5) compared to variants with a low impact on splicing (|ΔΨ| < 5) (Fig. 9a). Much of the conservation observed is likely due to protein coding constraints on sequences, which may add noise to this signal. To investigate whether this splicing-sensitive conservation is stronger in variants without protein changing potential, we examined the same trend in variants without protein coding constraints (intron, synonymous, UTR, and splice region variants), and we observed a more significant difference (Fig. 9b). Additionally, when we focus on synonymous variants only, the effect is much clearer, even with a smaller sample size (Fig. 9c). Intron variants seem to show the same trend of higher conservation with higher |ΔΨ|; however, it is a milder effect (Fig. 9d). This suggests that ESEs and ESSs modulated by these variants are more conserved, while intronic regulatory regions in the window we are testing are relatively more flexible. Perhaps this weaker conservation signal is because ISSs and ISEs are not constrained by the context of the protein frame, and may be able to move around in linear space within the intron and still be effective in influencing splicing.

**Fig. 10:**
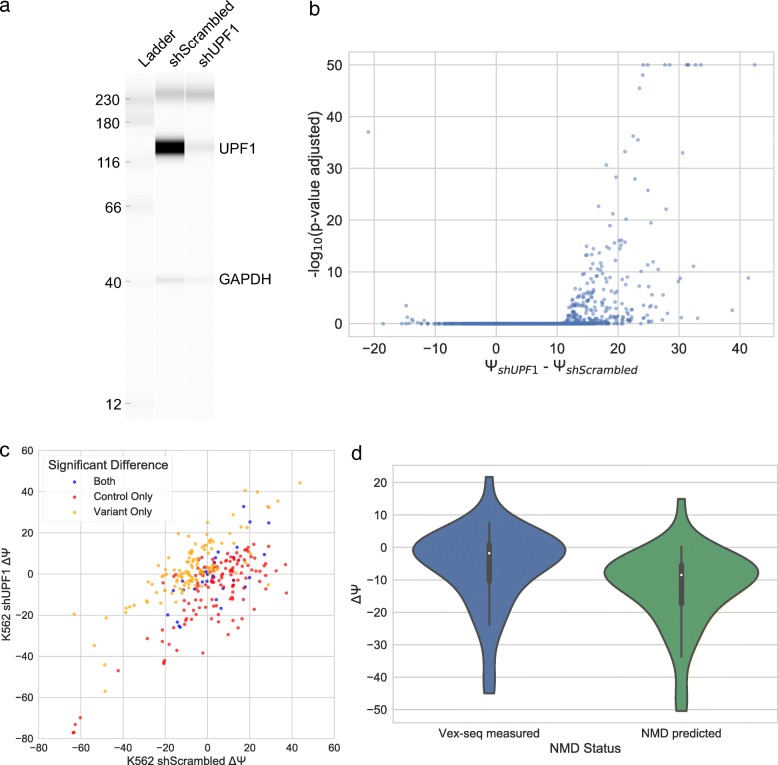
The impact of NMD on Vex-seq splicing results. **a** Western blot from Wes showing UPF1 knockdown in K562 cells. **b** A volcano plot showing the significance (assessed by rMATS-STAT) and change in splicing of shUPF1 cells compared to a scrambled control. **c** A scatter plot showing the behavior of ΔΨ of test exons in which there is a significant difference in the shUPF1 cells compared to the shScrambled cells. The color coding highlights test exons in which a significant difference in Ψ was identified. **d** Violin plots showing the predicted impact of NMD on variants which would be subject to NMD endogenously, but not in Vex-seq

One confounding variable for particular test exons in the context of Vex-seq is nonsense-mediated decay (NMD), which may be different from NMD in the endogenous context. To investigate the role of NMD in this assay, we used a *UPF1*-targeted shRNA knockdown to attenuate NMD in the K562 cell line and performed the Vex-seq in this new context [23]. UPF1 protein was depleted 63% (Fig. 10a). To identify transcripts that may be sensitive to NMD, we performed a differential splicing analysis using rMATS-STAT [24]. As expected, most transcripts that are significantly changing display increased exon inclusion (Fig. 10b). NMD is known to act through premature termination codons (PTCs), which can be predicted based on the presence of a stop codon 50 nucleotides before the last exon junction in the transcript [25]. While not all test exons with PTCs have a significant increase in splicing upon UPF1 depletion, most (95/151) significantly changing (*p* ≤ 0.01) test exons have a predicted PTC in the context of Vex-seq. This allows us to identify transcripts which are NMD-sensitive in this experimental system, but not necessarily in the endogenous context (Fig. 10c). To characterize the effect of NMD in our assay, and how it would relate to the wild-type situation, we used linear regression to predict the effect of NMD on transcripts that would be endogenously subject to NMD, but may not necessarily be in Vex-seq. This model uses the UPF1 knockdown Ψ and the presence of a PTC as input to predict the shScrambled Ψ (mean squared error (MSE) = 69.81) and performs better than a model without PTC input (MSE = 80.22) on a 1/3 held out test set. The predicted effect of endogenous NMD on ΔΨ of stop gained and frameshift variants is shown in Fig. 10d.

## Discussion and conclusions

We have developed a new assay to assess how variants can impact pre-mRNA splicing efficiency called Vex-seq. This method builds upon previous high-throughput splicing reporter assays. It utilizes a barcoding approach and designed sequences based on the transcriptome and genetic variants. Vex-seq’s approach of using designed sequences allows for the possibility of not deeply sequencing the plasmid pool, because barcode variant associations are already known. This assay is also able to test designed intronic variation which other recent methods have been unable to do, until very recently [9, 26]. Vex-seq is even able to account for the impacts that variants may have on transcription of reporter transcripts because of the barcoding approach. Vex-seq could be applied to a number of different applications, including fine mapping of GWAS variants that may be involved in splicing regulation, which has been shown to be linked to complex diseases [3]. Additionally, this could be used to dissect the behavior of RNA binding proteins and their effect on splicing regulation, or even saturating mutagenesis of exons known to be important for diseases. Thus, Vex-seq has the potential to have an extremely high impact on our understanding of genome function and how non-coding sequence variants impact pre-mRNA splicing.

While Vex-seq offers certain advantages over current methods, there remain some obstacles with all of these splice reporter approaches [8, 9]. First, these massively parallel splicing assays lack the context of the entire gene and chromatin state of the native genes. Second, these assays have limitations in terms of barcode design and synthesis length constraints and also may have cryptic splice sites formed in the context of the mini-gene. As oligonucleotide synthesis technologies improve, more context can be added to exons tested in this way. With more context, we expect Vex-seq to be more accurate at identifying variants that impact splicing.

Despite only examining 110 alternative exons in this study, we are able to obtain some biological insights from these data. The first is the similarity between the splicing behavior of K562 and HepG2 cell lines. Although the precise Ψ of each exon variant is not necessarily identical between the two cell lines, the directionality of the ΔΨ induced by most variants is quite similar in each cell line (Fig. 5b). This may suggest that most variants tested in this context are acting upon splicing elements common across these cell lines. Of course there are exceptions to this behavior, which may mechanistically be related to the unique *trans* factors of each cell line or noise in the data. This observation may change when analyzing splicing changes in response to stimuli or in the context of a cell with more complex transcriptome regulation. Alternatively, this may suggest that regulatory factors important for cell type-specific splicing are generally outside of the window that we are testing in Vex-seq. The predictive power of conserved intronic splicing regulatory elements on Ψ generally being within 100 nucleotides upstream and downstream may suggest that this is the case [27]. We have also been able to use this assay after UPF1 depletion to account for NMD as an experimental artifact, but also use it to predict the impact of NMD on variants which would cause NMD endogenously.

Data obtained from Vex-seq demonstrate the importance of variants on impacting pre-mRNA splicing efficiency. It shows that variant effect prediction, while useful for predicting protein changing variants, is insufficient to predict all splicing changes induced by variants. We also show that variants that tend to change splicing more are also generally more conserved than nucleotides that do not, particularly when the variants are otherwise not predicted to change protein products.

## Methods

### Plasmid alterations

pcAT7-Glo1 was provided by Kristen Lynch. To eliminate a splice acceptor site in the middle of intron 1, a deletion of the pyrimidine tract and splice acceptor sequence was deleted. This was done through digestion of the vector with AflII and PstI and PCR amplifying an insert using two primers (FWD 5′-AAACTCTTAAGCTAATACGACTCACTATAGG-3′, REV 5′- GACTGAATGAGTCTGCAGAGGCAGAGAGAGTCAGTGG-3′). The insert digested with AflII and PstI was ligated in the vector digested with the same enzymes, resulting in the plasmid used for these studies.

### Assembly of Vex-seq plasmid

The oligo pool (Additional file 1: Table S1) was amplified with a common primer set (FWD 5′- GTAGCGTCTGTCCGTCTGCA-3′; REV 5′-CTGTAGTAGTAGTTGTCTAG-3′) for 20 cycles, then digested with PstI and XbaI. These were subcloned into the modified pcAT7-Glo1 also using PstI and XbaI sites. The resulting plasmid pool, referred to as 1˚, was then digested with SpeI and MfeI. Exon 3 and intron 2 were PCR amplified from the original plasmid with primers (FWD 5′-GTGTGGAAGTCTCAGGATCG-3′, REV 5′-AACGGGCCCTCTAGAGC-3′) and digested with MfeI and XbaI. The resulting product was subcloned into the digested 1˚ vector resulting in the final plasmid pool (2˚).

### Transfection and cell culture

HepG2 cells were grown to a density of 0.5 × 10^6^ cells per well and transfected with 1 μg of plasmid DNA using Lipofectamine 2000. Transfected HepG2 cells were then selected with 1 mg/mL zeocin for 8 days. K562 cells were grown to a density of 1.0 × 10^6^ cells per well and electroporated with 5 μg of plasmid DNA. Transfected K562 cells were then selected with 200 μg/mL of zeocin for 8 days. RNA from each cell line was isolated using Maxwell® 16 LEV simplyRNA Purification kits.

UPF1 knockdown experiments were performed by transducing K562 cells with shRNA TRCN0000022254 (TRC collection), hairpin sequence (5′-CCGG-GCATCTTATTCTGGGTAATAA-CTCGAG-TTATTACCCAGAATAAGATGC-TTTTT-3′). A scrambled shRNA (SHC002 Sigma-Aldrich; 5′-CCGG-CAACAAGATGAAGAGCACCAA-CTCGAG-TTGGTGCTCTTCATCTTGTTG-TTTTT-3′) was used as a non-specific control. Transfected cells were selected with puromycin for 5 days followed by transfection with the Vex-seq plasmid library. Cells were then harvested after 24 h and RNA was collected as above. Protein was isolated and western blotting performed using Wes.

### Sequencing preparation

Sequencing for the 1˚ library was constructed using a nested PCR reaction. The 1˚ library was amplified for 15 cycles using the following primers: FWD 5′- ACACTCTTTCCCTACACGACGCTCTTCCGATCTCCACTGACTCTCTCTGCCTC-3′; REV 5′-GTGACTGGAGTTCAGACGTGTGCTCTTCCGATCTAGCGGGTTTAAACGGGCCCT-3′. The 2˚ library was amplified for 15 cycles using the following primers: FWD 5′- ACACTCTTTCCCTACACGACGCTCTTCCGATCTAGCAGCTAAATCCAGCTACCA-3′; REV 5′-GTGACTGGAGTTCAGACGTGTGCTCTTCCGATCTAGCGGGTTTAAACGGGCCCT-3′. Each of these products was then amplified for ten cycles using the following primers: FWD 5′-AATGATACGGCGACCACCGAGATCTACAC*-i5-INDEX-*ACACTCTTTCCCTACACGACGCTCTTCCGATCT-3′; REV 5′-CAAGCAGAAGACGGCATACGAGAT*-i7-INDEX-*GTGACTGGAGTTCAGACGTGTGCTCTTCCGATCT-3′. The cDNA was synthesized from the K562 and HepG2 RNA using SuperScript™ III reverse transcriptase and a gene specific primer (5′-GTGACTGGAGTTCAGACGTGTGCTCTTCCGATCTNNNNNNNNNNGCAACTAGAAGGCACAGTCGAGG-3′). The cDNA was then PCR amplified for ten cycles using the following primers: FWD 5′-AATGATACGGCGACCACCGAGATCTACAC*-i5-INDEX-*ACACTCTTTCCCTACACGACGCTCTTCCGATCTGGCAAGGTGAACGTGGATGAAG-3′; REV 5′-CAAGCAGAAGACGGCATACGAGAT*-i7-INDEX-*GTGACTGGAGTTCAGACGTGTGCTCTTCCGATCT-3′. Resulting samples were multiplexed and sequenced on a MiSeq using a v2 300-cycle kit. Read 1 and read 2 were 150 bases each.

### Data analysis and interpretation

#### Plasmid quality control

Forward and reverse reads from plasmids were combined into a single read using FLASH [28]. The 1˚ library reads were sorted into bins using the barcode and grouped by control exon backbone with separate bins for indels and control sequences. Reads were then aligned using Novoalign V3.02.13 (http://www.novocraft.com). Sam2tsv was then used to identify variants in each read and identify the barcode sequence [29]. Barcodes with 15% or more of reads not containing the correct variant were filtered out during splicing analysis using custom python scripts. Barcodes identified 2˚ library reads using custom python scripts and barcodes without reads were filtered out of the analysis.

#### Splicing alignments and analysis

Reads were identified by barcode and sorted into bins for each variant. The duplicate reads in each bin were then collapsed into a single read by the UMI. Reads were then aligned to a variant-specific reference using STAR version 2.5.2b [13]. The uniquely aligned annotated read junctions were identified and Ψ and ΔΨ were calculated. Reads which spanned unannotated splice junctions were discarded for calculating Ψ and ΔΨ. Ψ values for analysis, unless otherwise indicated, were the mean of the K562 and HepG2 Ψ values. Mutated and consensus splice site controls were removed for most analyses with the exceptions of Figs. 2 and 4. Annotations for each variant were done using the Ensembl Variant Effect Predictor tool using assembly GRCh37.p13 and the Ensembl transcript database [20]. The variants used in the analysis were selected based on the first annotation output by VEP. We used 100-way vertebrate PhyloP conservation scores to examine conservation [22]. Scripts to reproduce the post-processed data can be found at https://github.com/scottiadamson/Vex-seq.

## Additional files

Additional file 1: This document contains **Table S1.**, which kists the oligo sequences used to assemble the splicing reporters along with their corresponding variants. (XLSX 588 kb)

Additional file 2: Reviewer reports and Author’s response to reviewers. (DOCX 87 kb)
