# Supplementary material for: Vex-seq: high-throughput identification of the impact of genetic variation on pre-mRNA splicing efficiency
Source: Genome Biol. 2018 Jun 1;19:71. doi: 10.1186/s13059-018-1437-x (PMC5984807; doi:10.1186/s13059-018-1437-x)
Supplement: Supplementary file 2 — Reviewer reports and Author’s response to reviewers. (DOCX 87 kb) [file 13059_2018_1437_MOESM2_ESM.docx]

**First round of review**

**Reviewer 1**

**Are the methods appropriate to the aims of the study, are they well described, and are necessary controls included?**

Yes

**Are the conclusions adequately supported by the data shown?**

Yes

**Are sufficient details provided to allow replication and comparison with related analyses that may have been performed?**

Yes

**Does the method perform better than existing methods (as demonstrated by direct comparison with available methods)?**

Yes

**Is the method likely to be of broad utility? Is any software component easy to install and use?**

This is an advance in a few ways. 1) it uses actual exons and known variants rather than artificial random sequences, 2) it can account for variants within both exon and intron sequences, 3) it provides a highly parallel and high throughput method.

**Is the paper of broad interest to others in the field, or of outstanding interest to a broad audience of biologists?**

No

**Comments to authors**

In this paper, Graveley and colleagues continue a theme that has been examined previously using a variety of tools. We know that splicing choices are made in the context of cellular machinery interacting with primary and secondary splicing signals and we know that mutations to these signals can have significant effects on splicing. However, we have a fairly poor ability to predict the splicing effect of most mutations or variants. This has consequences particularly in the context of genomic medicine in which distinguishing significant changes from background is important.

Here, the authors implement a new method for designing, constructing and evaluating the function of a set of exons and variants in a three exon minigene construct. They used 110 human exons and 2059 known variants as their starting point. Using a parallel and high throughput approach called vex-seq, they measured the splicing efficiency of each exon and the change in splicing efficiency due to each variant in two different human cell lines. 

The technique is clever and the results appear to be robust in the technical sense with good reproducibility. The authors demonstrate that some variants are able to reduce or enhance exon inclusion substantially. The summary figure 6 shows the distribution of effects in a metagene format. From this, one can observe that the largest negative effects are due to variants that affect the immediate 5' and 3' splice sites as well as the putative polypyrimidine tract region. Other exonic regions showing effects are described as " Evidence of potential ESS and ESE regulatory elements can be observed within the exon, as variants in the exon are capable of inducing strong ΔΨ changes in either directions." It is not clear if the authors made an attempt to relate specific ESS or ESE motifs to the effects they see. To my mind, this kind of information would be a significant addition to the field. 

Another concern with the approach is how to account for nonsense mediated decay. The authors note that "these approaches also have some difficulty accounting for nonsense mediated decay, which may bias different variants compared to their impact in the context of the transcriptome." In fact, their method is likely to reveal a complex mixture of effects due to splicing and differential stability of transcripts due to NMD. These could be separated by either using cell lines with reduced NMD activity and/or incorporating a stability determination in addition to the splicing determination. 

Overall, this is a potentially useful technique but the results as presented seem less impressive or informative than they could be. In addition, the NMD issue further clouds the interpretation. 

Minor points:
Figure 3 needs an expanded legend. It is not easy to figure out what is being measured. In addition, the axis labels are too small. Perhaps, the two panels could be displayed vertically to allow a larger size.

Figure 6: "Variant" is misspelled. 

Figure 6: A useful addition to this figure would be a track showing the number of variants sampled at each position. 

Figure 7: it is not clear as to the difference between "splice region" and "5' splice site" and "3' splice site".

**Reviewer 2**

**Are the methods appropriate to the aims of the study, are they well described, and are necessary controls included?**

See my comments below

**Are the conclusions adequately supported by the data shown?**

See my comments below

**Are sufficient details provided to allow replication and comparison with related analyses that may have been performed?**

See my comments below

**Does the method perform better than existing methods (as demonstrated by direct comparison with available methods)?**

No: see my comments below

**Is the method likely to be of broad utility? Is any software component easy to install and use?**

See my comments below

**Is the paper of broad interest to others in the field, or of outstanding interest to a broad audience of biologists?**

No

**Comments to author**

High-Throughput Identification of Genetic Variation Impact on pre-mRNA Splicing Efficiency

In this paper, the authors develop VEX-seq to identify genetic variations that mediate alternative splicing. This approach is based on massively parallel mini-gene reporter assay allowing detection of both exon inclusion and exclusion. The minigene plasmid vector is designed for rapid cloning and assessment of the impact of sequence variants on splicing. Therefore, this method is potentially useful to interpret the numerous sequence variants of unknown biological and clinical significance found in genetic screenings, confronting a major challenge in the molecular diagnosis of genetic disease including cancer susceptibility. 

Major points
1. The authors claimed that the minigene reporter used for Vex-seq is capable of testing both exonic and intronic variants, which makes this paper distinguishable from the recent study (Soemedi et al. 2017, Nat. Genet.). As claimed, there is room for introducing an intronic variant to the minigene reporter. However, it is very restricted in size, implicating that very limited cases of intronic variants can be tested. Therefore, the authors at least should put an effort to demonstrate validity of the minigene reporter system with known ISE/ISS. And also it would be recommended to provide the genomic location of known and predicted intronic elements from 5' and 3' splice site. 

2. The minigene reporter system seems to be responding to very stringent perturbation (e.g. on 5' and 3' splice site) for splicing. To determine the sensitivity of the reporter system, the author needs to test various type of known exonic/intronic variants that altered splicing. 

3. The authors should provide the rationale for testing K562 and HepG2 cell lines with the minigene reporter. In addition, the correlation analysis between these two cell lines is not fully supportive of the conclusion of Figure5. 

Minor points
- General information of the MiSeq dataset should be provided. e.g. Read length
- The figure legends are not fully descriptive for the figures. 

Typo
- The middle panel in Figure 1a: XbaI -> SpeI

**Authors’ response to reviewers**

*We thank the reviewers for their thoughtful and constructive comments regarding our manuscript. We have now added additional new experiments, analysis and text in an attempt to address the issues raised by the reviewers. We believe that the paper is now much stronger and that we have addressed the issues. Below we outline the responses to the authors comments. The bold text highlights the main issues raised by the reviewers and our responses are indented and in italics:*

Reviewer #1: In this paper, Graveley and colleagues continue a theme that has been examined previously using a variety of tools. We know that splicing choices are made in the context of cellular machinery interacting with primary and secondary splicing signals and we know that mutations to these signals can have significant effects on splicing. However, we have a fairly poor ability to predict the splicing effect of most mutations or variants. This has consequences particularly in the context of genomic medicine in which distinguishing significant changes from background is important.

Here, the authors implement a new method for designing, constructing and evaluating the function of a set of exons and variants in a three exon minigene construct. They used 110 human exons and 2059 known variants as their starting point. Using a parallel and high throughput approach called vex-seq, they measured the splicing efficiency of each exon and the change in splicing efficiency due to each variant in two different human cell lines.

The technique is clever and the results appear to be robust in the technical sense with good reproducibility. The authors demonstrate that some variants are able to reduce or enhance exon inclusion substantially. The summary figure 6 shows the distribution of effects in a metagene format. From this, one can observe that the largest negative effects are due to variants that affect the immediate 5' and 3' splice sites as well as the putative polypyrimidine tract region. **Other exonic regions showing effects are described as "Evidence of potential ESS and ESE regulatory elements can be observed within the exon, as variants in the exon are capable of inducing strong ΔΨ changes in either directions." It is not clear if the authors made an attempt to relate specific ESS or ESE motifs to the effects they see. To my mind, this kind of information would be a significant addition to the field.**

*We thank the reviewer for raising this issue. We have added new analyses to address this issue on page 7 in the manuscript and Figure 7. We utilized ESS and ESE regulatory elements identified using the ESEseq dataset from Ke et. al 2011, which examines hexamer capacity to change splicing. We show in the revised manuscript that if we quantify the sum of the ESEseq hexamer scores across each exon and compare the variant and the reference sequence ESEseq scores, they associate well with the changes in splicing. If we examine each hexamer and add the ΔΨ for each variant which gains that hexamer, and subtract ΔΨ for each variant which loses that hexamer (compared to reference) and compare it to the ESEseq score for that hexamer, there is a weak but statistically significant correlation. The weak magnitude of the correlation is probably a consequence of the fact that many hexamers are represented by few variants as the library was not designed to explore ESE and ESS hexamer sequence space, but rather naturally occurring variants regardless of whether they overlapped potential ESE or ESS sequences).*

**Another concern with the approach is how to account for nonsense mediated decay. The authors note that "these approaches also have some difficulty accounting for nonsense mediated decay, which may bias different variants compared to their impact in the context of the transcriptome." In fact, their method is likely to reveal a complex mixture of effects due to splicing and differential stability of transcripts due to NMD. These could be separated by either using cell lines with reduced NMD activity and/or incorporating a stability determination in addition to the splicing determination.**

*We agree with the reviewer on this and have now added a new set of experiments in which we performed Vex-seq in UPF1 depleted K562 cells (using anti-UPF1 shRNA), which should have reduced NMD (Page 11, figure 10). We were able to identify exon variants that were differentially spliced between the control and UPF1 KD cells using rMATS-STAT. This allowed us to identify transcripts where NMD impacts splicing and calculate an NMD adjusted ΔΨ. As expected, these results do show that for transcripts that are sensitive to NMD, stronger splicing changes are observed in cells with reduced NMD. We do point out, however, that because the exons are tested in a mini-gene context, in many cases the frame of the exon in the mini-gene differs from that in the endogenous gene. Thus, exons that are NMD sensitive in the reporter may not be NMD sensitive in the endogenous context, and vice versa. We therefore also used this information and a linear regression to predict the impact of variants on splicing of exons where NMD would be active in endogenous context.*

Overall, this is a potentially useful technique but the results as presented seem less impressive or informative than they could be. In addition, the NMD issue further clouds the interpretation.

*We agree with the reviewer that this technique is useful. We point out that we formulated this manuscript as a methods paper that is primarily focused on describing the approach and demonstrating that it works and is reproducible, rather than providing a large amount of new information. We are in the process of using this approach in follow up studies that are specifically designed to address important biological issues related to the role of sequence variants involved in pre-mRNA splicing. We believe that the method alone is worth publication so that others in the field can use it to address issues of interest to them.*

Minor points:

**Figure 3 needs an expanded legend. It is not easy to figure out what is being measured. In addition, the axis labels are too small. Perhaps, the two panels could be displayed vertically to allow a larger size.**

*We have enlarged the axis labels and revised the figure legend as suggested.*

**Figure 6: "Variant" is misspelled.**

*We have corrected the spelling.*

**Figure 6: A useful addition to this figure would be a track showing the number of variants sampled at each position.**

*We have added a histogram to the top of Figure 6 to identify the number of variants plotted at each position.*

**Figure 7: it is not clear as to the difference between "splice region" and "5' splice site" and "3' splice site".**

*We have made this clearer in the figure legend.*

Reviewer #2: High-Throughput Identification of Genetic Variation Impact on pre-mRNA Splicing Efficiency

In this paper, the authors develop VEX-seq to identify genetic variations that mediate alternative splicing. This approach is based on massively parallel mini-gene reporter assay allowing detection of both exon inclusion and exclusion. The minigene plasmid vector is designed for rapid cloning and assessment of the impact of sequence variants on splicing. Therefore, this method is potentially useful to interpret the numerous sequence variants of unknown biological and clinical significance found in genetic screenings, confronting a major challenge in the molecular diagnosis of genetic disease including cancer susceptibility.

Major points

1. The authors claimed that the minigene reporter used for Vex-seq is capable of testing both exonic and intronic variants, which makes this paper distinguishable from the recent study (Soemedi et al. 2017, Nat. Genet.). As claimed, there is room for introducing an intronic variant to the minigene reporter. **However, it is very restricted in size, implicating that very limited cases of intronic variants can be tested. Therefore, the authors at least should put an effort to demonstrate validity of the minigene reporter system with known ISE/ISS. And also it would be recommended to provide the genomic location of known and predicted intronic elements from 5' and 3' splice site.**

*The reviewer makes a good point in that ISSs and ISEs have typically been studied further downstream from the exon than what we have studied in our experimental system (Wang et. al, 2012; Culler et. al, 2010). We have modified the text accordingly when discussing ISEs and ISSs to more directly and accurately address this point. However, we do observe many variants upstream of the exon which do change splicing and are outside of the window tested for 3’ splice site maximum entropy. We have attempted to look at variants which change splicing branchpoints with Branchpointer (Signal et. al, 2017), however we find that most variants upstream of the 3’ splice site region measured for maximum entropy that impact splicing (53/84 with  |ΔΨ| > 5) do not change branchpoint usage probability or branchpoint number (shown below), and therefore could be ISS or ISE elements.*

*Nine variants with  |ΔΨ| > 5, have a change in branchpoint number (Response Figure 1) and 31 have a change in branchpoint usage probability (Response Figure 2).*

*Response Figure 1. Plot showing ΔΨ for variants > 20 bases upstream of the 3’ splice site and that change splicing ( |ΔΨ| >5) and predicted change in branchpoint number from Branchpointer. Note that none of the number of predicted branchpoints go to zero with genetic variants. There is no significant correlation.*

*Response Figure 2. Plot showing ΔΨ for variants > 20 bases upstream of the 3’ splice site and that change splicing ( |ΔΨ| >5) and predicted change in predicted maximum branchpoint probability from Branchpointer. There is no significant correlation.*

*We also examined whether secondary structure around the 3’ splice site could explain these variant as has been suggested recently (Soemedi et al., 2017), but did not find any obvious correlation or explaination (Response Figure 3).*

*Response Figure 3. Plot showing ΔΨ for variants > 20 bases upstream of the 3’ splice site and the change in base pair probability for the 3’ splice site dinucleotides. There is no significant correlation.*

**2. The minigene reporter system seems to be responding to very stringent perturbation (e.g. on 5' and 3' splice site) for splicing. To determine the sensitivity of the reporter system, the author needs to test various type of known exonic/intronic variants that altered splicing.**

*We agree with the reviewer that calibrating the reporter to various types of known variants would be ideal. However, we are not aware of any set of gold standard sequences where it is possible to accurately predict the magnitude of change in splicing that mutating one hexamer at a specific location in the exon or intron to another would have in this context. This is further compounded by the fact that basal Ψ values do fluxuate depending on the cell line being used. For this reason, we opted to design and analyze the fully mutated and consensus control splice site sequences in the system, precisely because they do have more or less a binary behavior. We believe that new our analysis of the impact of the exon and intronic variants from ExAC that we tested, and how these relate to changes in splicing as a function of changes in splice site maximum entropy and predicted ESS and ESE sequences, demonstrates the sensitivity of the reporter system (Page 8 and Figure 7). We also reiterate that this manuscript is intended to serve as a methods paper and that future studies will be focused on specific classes of splicing elements that will facilitate further insights into the sensitivity of the system.*

**3. The authors should provide the rationale for testing K562 and HepG2 cell lines with the minigene reporter.**

*We chose these two cell lines for this study because our laboratory is using them extensively as part of the ENCODE project. In addition, these cell lines are derived from two different tissue types and express various RBP encoding genes at different levels. This design allowed us to demonstrate that most variants impact splicing in the same direction regardless of cell type.*

4. **In addition, the correlation analysis between these two cell lines is not fully supportive of the conclusion of Figure 5.**

*We apologize if our description of these results was not clear. We don’t argue, or even expect, that the splicing behavior of these variants in the two cell lines are exactly the same, we argue that they are similar. We believe that this is supported by the high correlation coefficients of ΔΨ between the two cell lines and the directionality agreement between the two cell lines (|ΔΨ| >5 at 92.61% and |ΔΨ| >10 at 97.49%). We have adjusted the writing on page 7 and revised Figure 5B to more clearly make this point.*

Minor points

**- General information of the MiSeq dataset should be provided. e.g. Read length**

*We have clarified this at the end of the “Sequencing Preparation” section in the Methods.*

**- The figure legends are not fully descriptive for the figures.**

*This issue was also raised by reviewer 1 and we have adjusted some of them accordingly.*

Typo

**- The middle panel in Figure 1a: XbaI -> SpeI**

*This isn’t actually a typo; they are different restriction sites that can be ligated due to their compatible ends.*

Citations:

Ke S, Shang S, Kalachikov SM, Morozova I, Yu L, Russo JJ, et al. Quantitative evaluation of all hexamers as exonic splicing elements. Genome Res. 2011;21:1360–74.

Shen S, Park JW, Lu Z, Lin L, Henry MD, Wu YN, et al. rMATS: robust and flexible detection of differential alternative splicing from replicate RNA-Seq data. Proc. Natl. Acad. Sci. U. S. A. 2014;111:E5593-5601.

Wang Y, Ma M, Xiao X, Wang Z. Intronic splicing enhancers, cognate splicing factors and context-dependent regulation rules. Nat. Struct. Mol. Biol. 2012;19:1044–52.

Culler SJ, Hoff KG, Voelker RB, Berglund JA, Smolke CD. Functional selection and systematic analysis of intronic splicing elements identify active sequence motifs and associated splicing factors. Nucleic Acids Res. 2010;38:5152–65.

Signal B, Gloss BS, Dinger ME, Mercer TR. Machine learning annotation of human branchpoints. Bioinforma. Oxf. Engl. 2017

Soemedi R, Cygan KJ, Rhine CL, Glidden DT, Taggart AJ, Lin C-L, et al. The effects of structure on pre-mRNA processing and stability. Methods San Diego Calif. 2017;125:36–44.

**Second round of review**

**Reviewer 1**

I am satisfied with the changes and recommend publication.
